# Salvage treatment of acute respiratory failure after autogenous tissue flap transplantation for chronic empyema with chest wall sinus: a case report and literature review

**DOI:** 10.1186/s13019-024-02488-2

**Published:** 2024-01-30

**Authors:** Lei Wang, Fei Chen, Zhongliang He, Xueming He, Chun Zhang

**Affiliations:** 1 Department of Cardiothoracic Surgery, Tongde Hospital of Zhejiang Province, 234 Gucui Rd, Hangzhou, China; 2 Department of Traumatology and Orthopedic Surgery, Tongde Hospital of Zhejiang Province, 310012 Hangzhou, Zhejiang China

**Keywords:** Empyema, Pneumonectomy, Free myocutaneous flap, Respiratory failure

## Abstract

**Background:**

Chronic empyema with chest wall sinus is a difficult and complex disease caused by multiple causative factors. It is difficult to control local infection due to its possible combination of bronchopleural fistula (BPF) and residual bone.The relevant literature emphasizes some risk factors for empyema progression after pneumonectomy, while the correlation between empyema and BPF after pneumonectomy increases mortality by infecting the remaining lungs. After pneumonectomy, the lung function of the contralateral side is particularly important.

**Case presentation:**

This paper reports a 62-year-old male patient who underwent right pneumonectomy for squamous cell carcinoma of the lung 12 years ago and began to develop empyema with anterior chest wall sinus 3 years ago. After admission, chest computed tomography (CT) showed right pleural effusion and formation of chest wall sinus. According to his clinical symptoms and imaging examination, he was diagnosed as chronic empyema with chest wall sinus.Due to the huge residual cavity of the patient,the clinical effect of using free vastus lateralis myocutaneous flap combined with pedicled pectoralis major muscle flap to fill the abscess cavity was satisfactory,but acute respiratory failure occurred due to left lung aspiration pneumonia after operation.

**Conclusions:**

After a series of treatment measures such as tracheal cannula, tracheotomy, anti-infection, maintenance of circulatory stability, and rehabilitation training, the patient was ultimately rescued and cured. Postoperative follow-up showed that the muscle flaps survived and empyema was eliminated.

## Introduction

The treatment of chronic empyema with chest wall sinus is very complicated, especially those caused by post-pneumonectomy. Some patients even include a huge abscess cavity due to the inconspicuous collapse of the affected side of the chest,which often leads to poor infection control, more complications, and a high mortality rate [1–3]. These often require comprehensive treatment methods, including technique of thoracoplasty, management of BPF and abscess cavity, degree of infection control, and optimization of general conditions [4]. For patients with chronic empyema with huge abscess cavity, it is often impossible to completely eliminate the abscess cavity and achieve a curative effect. They often require long-term drainage with chest tubes, leading to poor quality of life. In addition to the initial thoracoplasty used to reduce the residual cavity and control infection, compound muscle flaps are one of the main surgical procedures for the treatment of chronic empyema, postoperative concerns include muscle flap activity and avoiding vascular crises. Acute respiratory failure caused by aspiration pneumonia is not common [5, 6].

We report a case of empyema with chest wall sinus after right pneumonectomy for squamous cell carcinoma. He developed acute respiratory failure after treatment with compound muscle flaps transplantation. After a series of treatment, he was finally rescued and cured.

## Case presentation

**Fig. 1:**
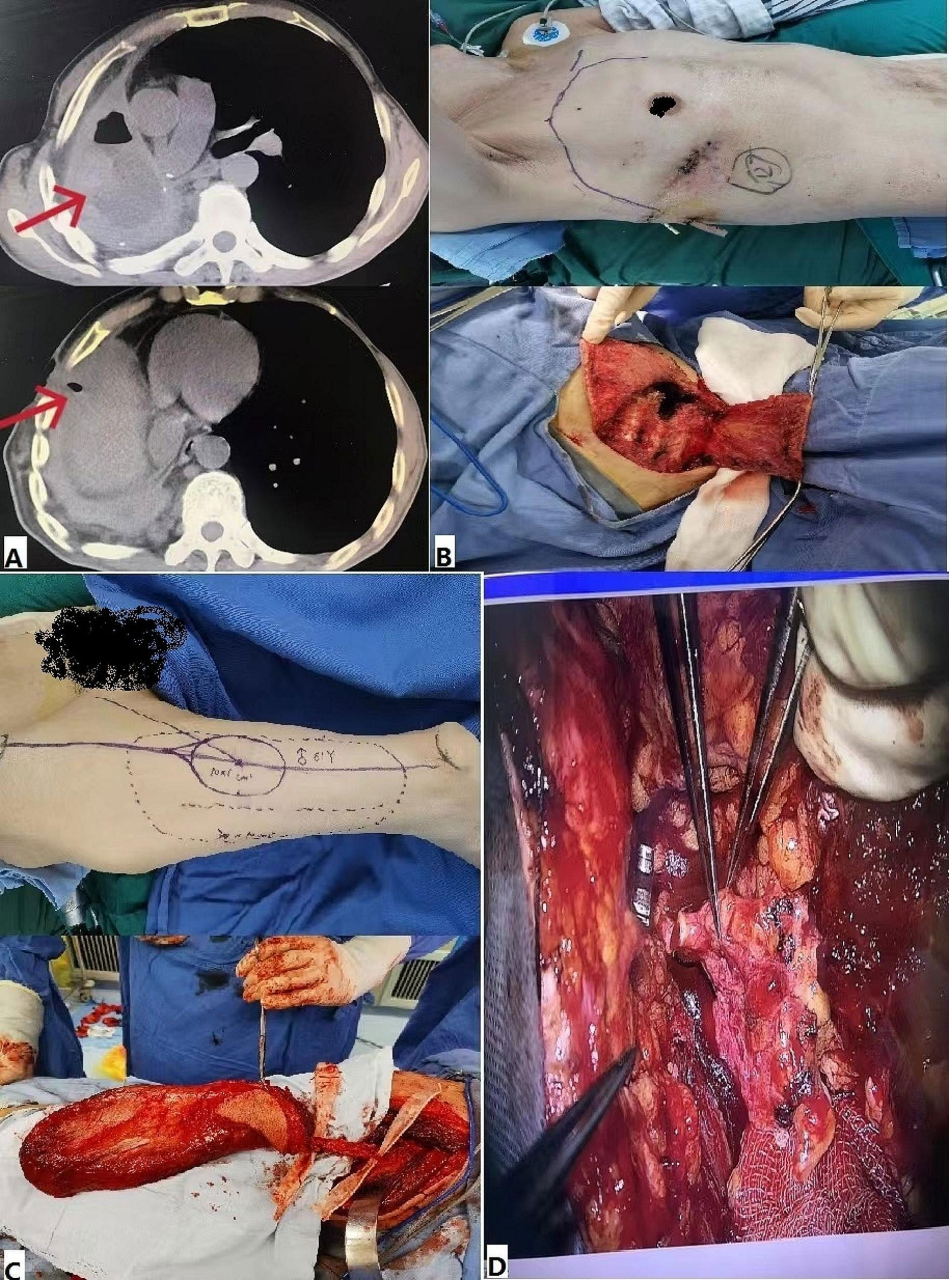
Preoperative and intraoperative clinical images of the patient. **(A)** Preoperative chest CT showed right encapsulated pleural effusion and pleural thickening (red arrow). The chest wall sinus was connected to the thoracic cavity (red arrow). **(B)** The design of preoperative chest incision and the pedicled pectoralis major muscle flap harvested during the operation. **(C)** Design of preoperative right lower limb incision and the vastus lateralis myocutaneous flap harvested during the operation. **(D)** Complete the anastomosis of vascular and nerve under the microscope

A 62-year-old male patient underwent right pneumonectomy under open-chest surgery after being diagnosed with poorly differentiated squamous cell carcinoma (central type) 12 years ago. He received 4 preoperative chemotherapy and 2 postoperative chemotherapy.He began to develop anterior chest wall sinus with pus discharge 3 years ago, and the main symptoms were cough and fever. During the illness, the main treatment was dressing changes on the wound surface of the sinus, which resulted in poor efficacy. Chest CT scan shows thickening of the right pleura, enveloping pleural effusion, and a sinus connecting to the abscess cavity can be seen on the right chest wall. Based on the patient’s clinical symptoms and imaging examination, we diagnosed a right chronic empyema with chest wall sinus. We resected the chest wall sinus and the right 5th and 6th rib segments in the first-stage operation to expose the abscess cavity. The necrotic tissue in the abscess cavity was removed and thoroughly debridement was performed. The volume of the abscess cavity was measured to be approximately 200 ml, and no bronchial stump fistula was found during intraoperative bronchoscopy and intrathoracic exploration. Two drainage tubes were left in the thoracic cavity and the incision was sutured. The pathogens identified by postoperative pus culture were *Pseudomonas aeruginosa and Klebsiella pneumoniae*. After 16 consecutive days of intrathoracic lavage (2000 ml of normal saline per day), sensitive antibiotic anti-infection, and nutritional support treatment, his symptoms significantly improved.When the patient met the following conditions postoperatively: ① Hemoglobin reached 100 g/L and plasma albumin reached 30 g/L;②The colour of drainage fluid became clear, and the drainage volume was less than 100 ml per day; ③ Re-pathogen culture negative; ④ The infection was initially controlled and the wound granulation was expected to be fresh. We chose radical treatment in second-stage operation. Debridement was first performed again in the second-stage operation. After debridement, the ipsilateral thoracodorsal artery, vein, and nerve were dissected in the right thoracic cavity as recipient vascular pedicles, and a pedicled pectoralis major muscle flap of approximately 12 cm × 8 cm was harvested. Then a free vastus lateralis myocutaneous flap of about 30 cm×10 cm in size was harvested from the patient’s right thigh (preserving the descending branch of the lateral circumflex femoral artery (LCFA), vein and nerve), and the size of the skin paddle was about 10 cm ×6 cm.The free vastus lateralis myocutaneous flap was transferred to fill the abscess cavity, and the lateral circumflex femoral artery, vein and nerve were anastomosed with the ipsilateral thoracodorsal vessels and nerve under the microscope with 9-prolene thread (Fig. 1). Finally, the skin paddle was sutured with the skin around the chest incision, and the subcutaneous drainage tubes were placed. Intraoperative blood transfusion included 1.5 units of suspended red blood cells and 210 ml of fresh frozen plasma. After operation, anti-infection, antispasm, anticoagulation and other treatments were given. The color of the skin paddle was closely observed and the right upper limb was immobilized [7].

**Fig. 2:**
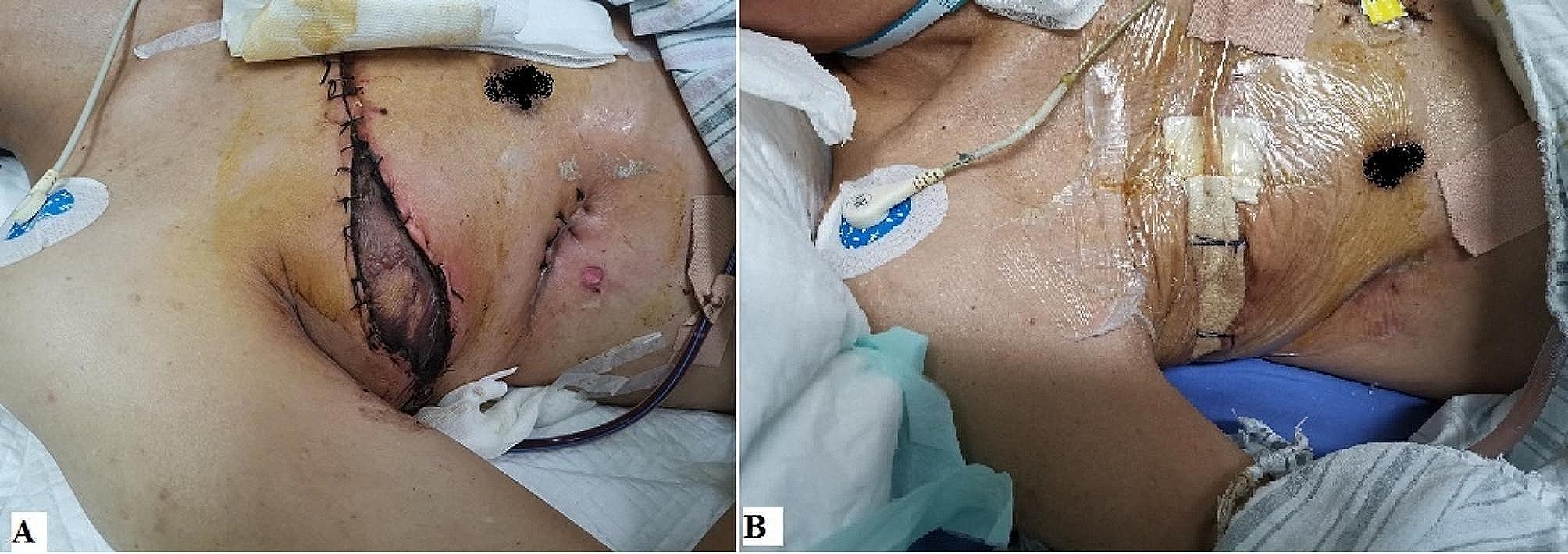
Postoperative complication and management of skin paddle. **(A)** Blisters were formed on the surface of the skin paddle and further ischemic necrosis was occurring. **(B)** After the necrotic skin paddle was removed, the wound was continuously flushing and drainage with VSD device

**Fig. 3:**
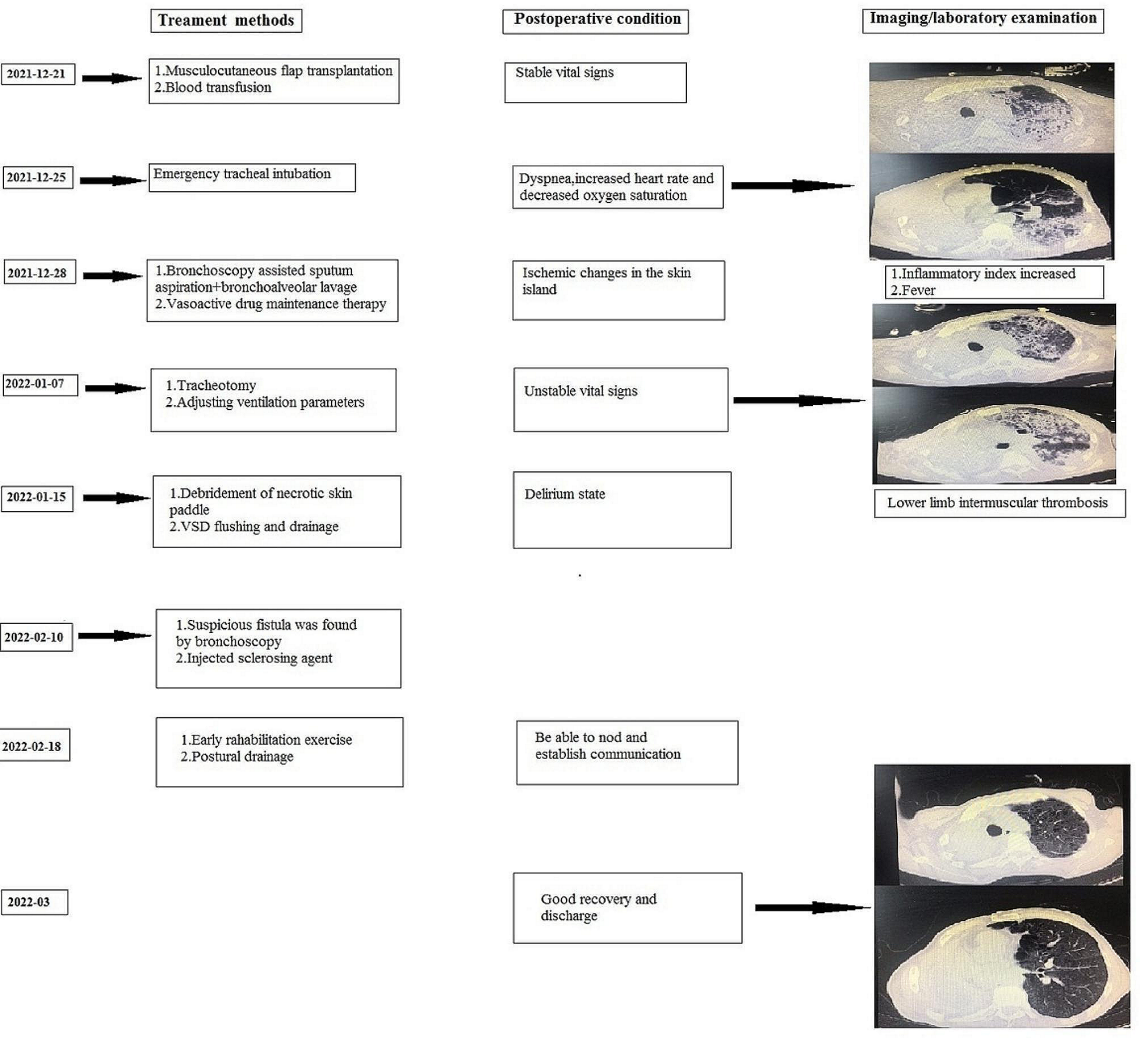
The treatment flowchart for the patient. The patient undergoes a series of treatment measures to obtain satisfactory results from surgery to recovery

**Fig. 4:**
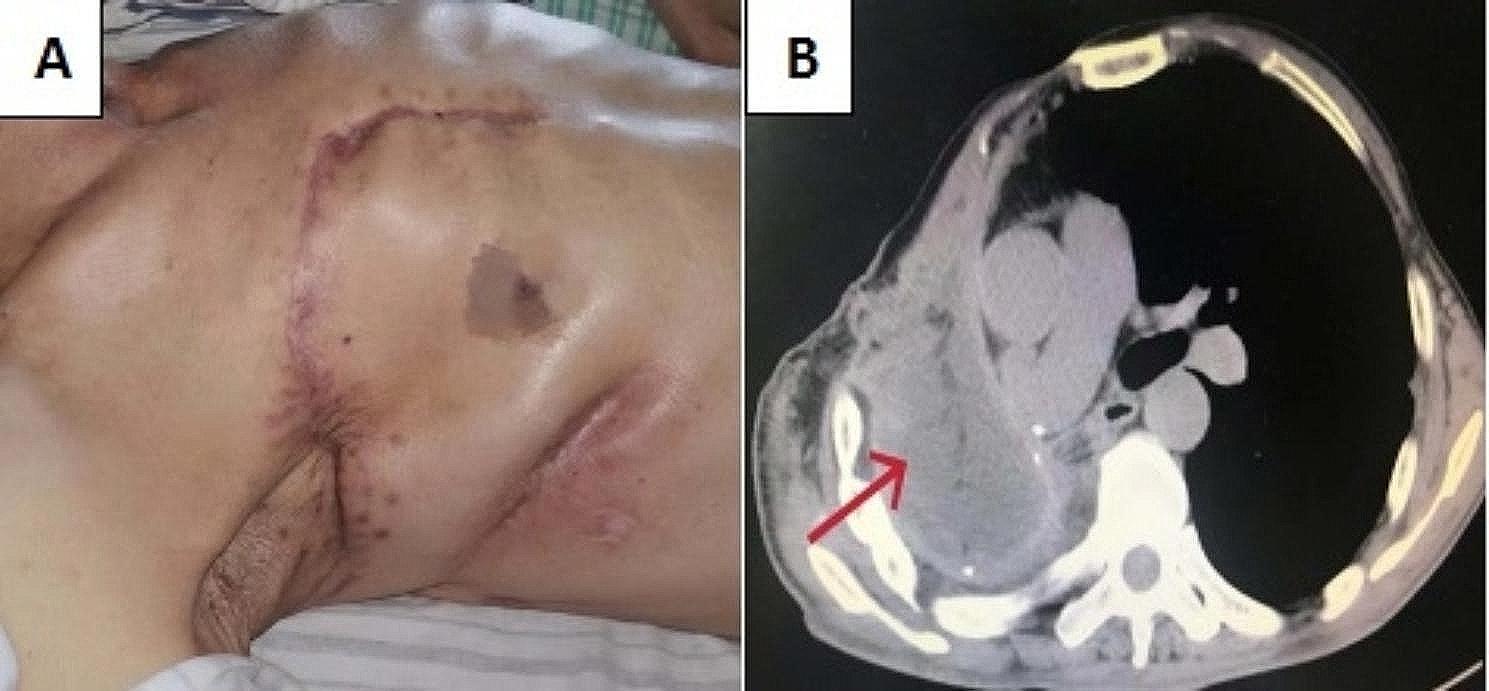
**(A)** Clinical data of postoperative patient. The postoperative chest incision recovered well. **(B)** Postoperative chest CT showed that the compound muscle flaps completely filled the abscess cavity, the empyema and chest wall sinus disappeared (red arrow)

The patient’s surgery was very smooth, with stable vital signs in the first three days after surgery and no vascular crisis or other complications. On the 4th day after operation, there was sudden dyspnea at night, with orthopnea and cyanosis. The auscultation of the left lung rale was not obvious, and the oxygen saturation under the mask was the lowest at 50%. The blood pressure was 102/60mmHg, and urgent blood related indicators were checked. D-dimer, B-type natriuretic peptide (BNP), and myocardial enzymes were normal or slightly higher. Ultrasound of blood vessels in both lower limbs showed no obvious thrombosis. The bedside chest X-ray indicates an increase in left lung markings. To shorten the rescue time, immediately intubate the bedside trachea and transfer to Emergency Intensive Care Unit (EICU) for further treatment. After the patient ‘s vital signs were stabilized, an urgent chest CT scan should be performed, and the left lung was patchy with increased density and multiple cavities with varying densities. Based on the patient’s symptoms, signs, and imaging examinations, we considered acute respiratory failure caused by aspiration pneumonia. Common respiratory system deteriorations caused by surgery include pneumonia caused by food inhalation and pneumonia caused by bronchopleural fistula (Further inquiry into the patient’s postoperative medical history revealed a small intake of water 3 hours before the condition worsened. The patient has strong ability to move independently, but the existing evidence is insufficient to support that food inhalation causes Pneumonia. Aspiration pneumonia in patients with bronchopleural fistula is often associated with changes in body position, which is a matter of concern). Given treatment such as tracheal intubation, sputum suction under bronchoscopy and intravenous injection of vasoactive drugs, it was still difficult to maintain respiratory and circulatory function. Therefore, we could not choose to have a tracheotomy and used a ventilator to assist breathing. Even worse, due to the prolonged use of vasoconstrictors, the skin paddle experienced necrosis 3 weeks after surgery. We removed the surface necrotic skin scabs and used vacuum-sealing drainage (VSD) devices for flushing and drainage (Fig. 2). After treatment such as anti-infection, posture drainage, sedation, and blood transfusion, pulmonary infection had slightly improved compared to before, and there were signs of improvement in inflammation related indicators. However, after the respiratory and circulatory system stabilized, there were signs of worsening pulmonary infection again, and respiratory failure was not significantly controlled. At this point, we had to reflect on whether there was a tracheal stump fistula? Airway nursing with fiberoptic bronchoscope was an important treatment link. When we performed the fifth deep sputum suction operation, we found suspicious small bubbles at the right tracheal stump, and promptly injected sclerosing agent under bronchoscopy to close the fistula. After a series of lengthy treatments mentioned above (lasting 153 days), the patient was ultimately rescued and discharged from the hospital (Fig. 3). Follow up for 8 months, chest CT scan showed survival of the right muscle flap, elimination of empyema, and disappearance of left lung infection (Fig. 4).

## Discussion

Post-pneumonectomy empyema (PPE) refers to obvious accumulation of pus or non-purulent fluid in the pleural cavity after surgery but the microbial culture is positive. Chronic empyema after pneumonectomy or lobectomy may be the result of a combination of factors. Common causes include postoperative BPF, untimely treatment of acute empyema, and secondary infection in the thoracic cavity [8]. Patients with chronic empyema with chest wall sinus require long-term drainage with tubes, especially after pneumonectomy with complications such as intrathoracic infection and BPF, which is a major challenge in current clinical treatment. The selection of surgical methods and timely management of postoperative complications are very important, especially in older patients. Timely treatment has a positive impact on the prognosis of critically ill patients such as muscle flap necrosis and respiratory failure [9, 10]. Respiratory failure caused by acute empyema is very common in clinical practice, and there have been reports of acute respiratory failure caused by some special bacteria [11]. However, acute respiratory failure caused by muscle flap transplantation after chronic empyema with chest wall sinus is rarely reported.

Patients undergoing pneumonectomy face the risk of decreased lung function and hemodynamic instability. Maintaining patient fluid balance is the key to fluid management after pneumonectomy. Excessive fluid intake may lead to pulmonary edema and breathing difficulties, while insufficient fluid intake may lead to dehydration and hemoconcentration. Therefore, personalized liquid management plans should be developed based on the specific situation of patients, including weight, intake and output, etc. Meanwhile, fluid management in post-pneumonectomy patients is crucial for preventing the development of acute respiratory distress syndrome (ARDS). Before and after surgery, a series of preventive measures should be taken, including quitting smoking, controlling blood sugar, and preventing infection. In addition, In addition, intraoperative tissue damage and bleeding should be minimized as much as possible to reduce the occurrence of postoperative pulmonary complications [12, 13]. When a patient with empyema after pneumonectomy underwent surgery again, he developed acute respiratory failure postoperatively. What do we need to think about? Acute pulmonary edema, acute lung injury(ALI), ARDS caused by postoperative fluid imbalance, or aspiration pneumonia caused by BPF? Respiratory failure caused by aspiration pneumonia is a common acute and severe condition, which often occurs in patients with long-term bedridden, severe trauma, shock after major surgery [14]. In this case, due to the experience of free vastus lateralis myocutaneous flap and pedicled pectoralis major muscle flap transplantation, which had caused great surgical trauma. In addition, the special posture relationship required after the operation, and at the same time, due to analgesia or anesthesia, the ability to protect the airway such as swallowing reflex and choking reflex was weakened or disappeared, the gastrointestinal function was weakened, and the risk of reflux related aspiration was increased. Of course, when suspicious air bubbles are detected through bronchoscopy, it is necessary to be highly vigilant about the possibility of tracheal stump fistula. If not intervened in a timely manner, postoperative exudate may reflux to the healthy side bronchus and cause inhalation pneumonia. Anyway, rescue treatment measures for acute respiratory failure are inevitable. Despite the increased risk of mechanical ventilation brought about by rescue treatment, prolonged hospitalization in EICU, inevitable use of antibiotics, and even the need for stepwise treatment or combination therapy [15], this successful rescue and cure case is still commendable for the patient with only one lung and who have suffered from extensive inhalation infections.

The management of PPE depends on the time of occurrence and whether there is a BPF, and the basic principles of abscess cavity management include closure of the BPF, optimal drainage of the residual cavity, and its subsequent filling. Of course, in order to minimize the recurrence of empyema after muscle flap transplantation, appropriate drainage methods such as partial rib resection and OWT are important prerequisite steps [16, 17]. The main goal of nursing in the acute phase is to protect the contralateral lung and control infection, which may take a considerable amount of time. The purpose of the late phase is to close the fistula and fill the abscess cavity [18]. Generally speaking, three weeks is the time point for distinguishing acute and chronic empyema. PPE usually occurs within 2 to 3 weeks, and bronchoscopy can confirm whether BPF exists. For empyema with BPF, because of the risk of continuous pus production, pleural cavity puncture and drainage must be carried out immediately. The most terrible complication is respiratory distress syndrome caused by aspiration pneumonia [19, 20]. Empyema without BPF may not have obvious clinical symptoms, and often forms a sinus in the skin tissue [18, 21] (Table 1).

**Table 1:** Management of pneumonectomy empyema

Objective	Acute phase(<3 weeks)	Late phase(>3 weeks)	
Infection control	① Drug anti-infectin + drainage with chest tube	①Empyema debridement + rib resection drainage	②Open window thoracostomy
	②Thoracoscopic debridement of empyema + drainage with chest tube	③drainage with chest tube	④Vacuum sealing drainage
Combined with BPF	Endoscopic closure of fistula		
Abscess cavity obliteration	After the infection was controlled,pull out the drainage tube or evaluate whether to eliminate the pus cavity through second-stage operation.	①Decortication of a fibrous pleura.	
		②Transplantation of tissue flaps such as muscle flaps, myocutaneous flap, and omental flaps.	

For patients with refractory chronic empyema with chest wall sinus, most of them have undergone multiple thoracic surgeries such as closed chest drainage, open-window thoracostomy (OWT), or thoracoplasty. Especially, the posterior lateral incision of the chest cuts off local muscles and vascular in the chest wall, making it difficult to harvest muscle flaps with sufficient volume nearby to eliminate the abscess cavity. Those with larger abscess cavity volumes are even more difficult [22–24]. Since 1989, it was reported that skeletal muscles outside the thoracic cavity were implanted into the thoracic cavity, and subsequent living tissues such as omental flap, rectus abdominis myocutaneous flap, and latissimus dorsi myocutaneous flap have also been clinically applied [25]. In terms of surgical method selection, we have observed that if the volume of the abscess cavity is less than 100mL, pedicled muscle flap or omental flap can be used for treatment. If the volume of the abscess cavity is between 100mL and 200mL, pedicled muscle flap, omental flap, free myocutaneous flap or compound muscle flaps can be used for treatment, which needs to be determined according to the intra-operative situation. For patients with a abscess cavity exceeding 200 ml, in order to completely eliminate the residual cavity, we generally prefer the treatment of free vastus lateralis myocutaneous flap. Although the surgical trauma is large, the tissue utilization rate is higher, and the abscess cavity is fully eliminated. In this case, we selected the vastus lateralis myocutaneous flap and pedicled pectoralis major muscle flap for the treatment of empyema. Compared with other muscle flaps, this tissue flap is easy to dissect, easy to harvest, large in size, and rich in muscle tissue, which can meet the requirements for filling huge cavity [26–29]. Besides, thoracoplasty is also an important step. Its purpose is to further reduce the abscess cavity, improve drainage, and create ideal conditions for later repair and reconstruction. In the past few years, standard thoracoplasty was often considered an abnormal surgery, and its indications were limited and not widely used. The reason is that most of the ribs, intercostal tissue and pleura need to be removed, which is a large surgical trauma and may even cause the chest wall to soften for a long time [30, 31]. However, for patients with chronic empyema who have a huge abscess cavity and cannot use pedicled muscle flap or other myocutaneous flap, we can choose to use free myocutaneous flap. In the first-stage surgery, 2–3 small segments of ribs are resected to improve drainage, and in the second-stage surgery, a free vastus lateralis myocutaneous flap is used to transfer and fill the abscess cavity. The utilization rate of autologous tissue is higher, and it does not need to be affected by the original chest incision and does not affect the appearance of the thorax.

Through the analysis and summary of this operation, we can not only focus on whether the reconstructed vessels are complicated with spasm, occlusion and other causes of tissue flap necrosis. Due to the occurrence of acute respiratory failure, we should reflect on its cause, because intraoperative no fistula of the tracheal stump was found, and the postoperative fiberoptic bronchoscopy was also verified several times,and only one of the examinations revealed suspicious signs. Is it the patient ‘s own aspiration or indeed a small fistula? Combined with this experience, the feasibility of intraoperative prophylactic use of sclerosants for closure of suspected fistulas in patients with the same condition requiring surgery next time is worth exploring.

In conclusion, the chest CT of this patient reexamined at postoperative follow-up showed no recurrence of empyema and chest wall sinus, and the transplanted compound tissue flap survived. Despite the acute respiratory failure in the postoperative period, the right grasp of the timing of rescue accumulated a certain amount of valuable experience for the adequate evaluation of the next surgery.

## Data Availability

Not applicable.

## Abbreviations

CT: Computed tomography
BPF: Bronchopleural fistula
LCFA: Lateral circumflex femoral artery
BNP: B-type natriuretic peptide
EICU: Emergency Intensive Care Unit
VSD: Vacuum-sealing drainage
PPE: Post-pneumonectomy empyema
ARDS: Acute respiratory distress syndrome
ALI: Acute lung injury
OWT: Open-window thoracostomy
